# Prospects of estrogen receptor β activation in the treatment of castration-resistant prostate cancer

**DOI:** 10.18632/oncotarget.16496

**Published:** 2017-03-23

**Authors:** Julia Gehrig, Silke Kaulfuß, Hubertus Jarry, Felix Bremmer, Mark Stettner, Peter Burfeind, Paul Thelen

**Affiliations:** ^1^ Institute of Human Genetics, University Medical Center Goettingen, Germany; ^2^ Department of Experimental Endocrinology, University Medical Center Goettingen, Germany; ^3^ Institute of Pathology, University Medical Center Goettingen, Germany; ^4^ Department of Neurology, University of Essen, Germany; ^5^ Department of Urology, University Medical Center Goettingen, Germany

**Keywords:** ADT, estrogen receptor β, 8β-VE2, androgen receptor, therapy resistance

## Abstract

Advanced prostate cancer can develop into castration-resistant prostate cancer (CRPC). This process is mediated either by intratumoral ligand synthesis or by mutations or aberrations of the androgen receptor (AR) or its cofactors. To date, no curative therapy for CRPC is available, as AR-targeted therapies eventually result in the development of resistance. The human prostate cancer cell line VCaP (vertebral cancer of the prostate) overexpresses AR and its splice variants (ARVs) as a mechanism of resistance to androgen-deprivation therapy (ADT) of external and intratumoral origin. In the present study, we demonstrate that stimulating estrogen receptor β activity with the specific agonist 8β-VE2 in VCaP cells in successive stages of ADT induced a time- and dose-dependent decrease in cell survival and an increase in apoptosis. Furthermore, 8β-VE2 treatment reduced the overexpression of the AR as well as ARVs in VCaP cells under maximum ADT. Our results indicate that decreased survival of the androgen-dependent CRPC cells employing apoptosis together with the regulative effect on AR expression could have beneficial effects over current AR-targeting therapies.

## INTRODUCTION

Prostate cancer is one of the most frequently diagnosed cancers and a leading cause of cancer-related deaths in Western society. For patients who develop progressive castration-resistant prostate cancer (CRPC), therapeutics targeting androgen receptor (AR) signaling have been proven to significantly prolong survival [1]. Regardless of previous treatments, novel therapies can still target AR activity that remains after preceding therapies. These new therapies include potent inhibitory AR-binding ligands, such as enzalutamide, or agents that exhaustively block androgen biosynthesis from all relevant sources, such as the selective inhibitor of CYP17A1, abiraterone. Still, the mechanisms implicated in the development of resistance to AR inhibition in prostate cancer are multiple and complex, involving virtually all classes of genomic alteration and leading to a host of selective/adaptive responses [2]. Therefore, even the most promising and highly effective new agents might eventually fail because of newly developed therapy resistance and subsequent failure. Moreover, efficient androgen deprivation and complete receptor blockade have not proven to be as complementary as anticipated or desired. This phenomenon of cross resistance became evident in studies of sequential or combinatorial use of these new individually effective drugs [3]. Two major aberrations were identified as the most likely underlying causes for the acquisition of therapy resistance and cross-resistance. The first is the occurrence of AR mutations, which lead to promiscuous employment of antiandrogens or other steroids for AR activation [4–7]. The second, which is observed more frequently, is the expression of different constitutively active AR splice variants [8]. Both mechanisms of resistance are observed after treatment with abiraterone as well as enzalutamide and result in the resumption of AR activity. Nevertheless, combinational therapeutic approaches targeting AR signaling and alternative oncogenic pathways are considered reasonable for patients with CRPC [9]. Taking into account that all approaches targeting the AR axis ultimately result in therapeutic failure, the search for alternative pathways becomes crucial.

Estrogen receptor β (ERβ, also known as ESR2) has been shown to have a role as a tumor suppressor in prostate cancer in various reports. The loss of ERβ expression correlates with increased proliferation in the ERβ-*knock out*/TRAMP (transgenic adenocarcinoma of mouse prostate) mouse model [10] as well as in human prostate cancer tumor tissue [11, 12]. Although ERβ is downregulated during prostate cancer progression [13], the activation or upregulation of ERβ inhibits tumor progression and induces cell cycle arrest and apoptosis in prostate cancer [14–16]. Our previous work focused on the activation of ERβ by subtype-specific ligands or by reversing the epigenetic silencing of histone modifications with the consequent anti-androgenic function of ERβ [17–19]. Therefore, AR downregulation by ERβ activation with subtype-selective ligands might achieve all-encompassing AR inhibition, including promiscuous gain-of-function AR mutations and splice variants acquired in earlier treatments. In addition, this concept should not by itself select for AR gain-of-function mutations or antiandrogen-to-androgen conversion because ERβ-targeted antiandrogen strategies do not involve inhibitory AR ligands with high affinity to AR. In this study, we evaluated the ERβ-selective ligand 8β-VE2 because of its antiandrogen features [20]. We used the prostate cancer bone metastasis cell model VCaP, which exists in three different stages of androgen-deprivation therapy (ADT) (androgen-sensitive, castration-resistant and under inhibition of intratumoral steroidogenesis by abiraterone acetate) to elucidate therapy sequencing options under obviation of cross resistance.

## RESULTS

### Generating different populations of VCaP cells

**Figure 1:**
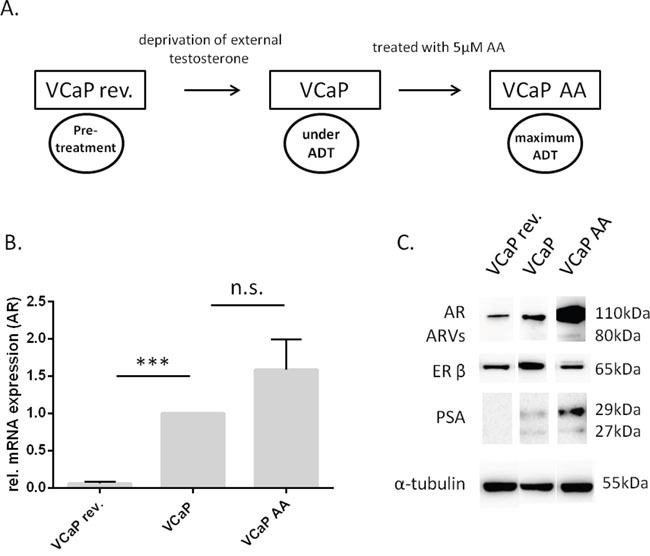
The VCaP variants differ in their expression of prostate cancer-relevant genes **(A)** VCaP rev were cultured with 1 nM testosterone over seven months, whereas VCaP AA were treated continuously with 5 μM abiraterone acetate. The three cells lines represent three different levels of androgen deprivation: VCaP rev, no therapy, subphysiological testosterone (0.3 ng/ml); VCaP, CRPC under first-line therapy (no detectable testosterone, data not shown); VCaP AA, CRPC under maximal, including intratumoral, ADT. **(B)** mRNA expression of the AR was measured by quantitative RT-PCR in all VCaP cell variations. AR expression in VCaP rev cells was significantly lower compared with that in VCaP control cells. AR mRNA expression in VCaP AA cells was not significantly higher than in VCaP control cells. The experiment was performed in triplicate. * P<0.05, ** P<0.01, *** P<0.0001, n.s. (not significant) compared with VCaP cells. **(C)** Representative western blot showing the three VCaP populations, VCaP rev, VCaP, and VCaP AA. Total protein was extracted, and immunoblots were probed with AR-specific, ERβ-specific, and PSA-specific antibodies. In VCaP rev cells, AR and PSA expression levels were remarkably lower than in VCaP cells. In VCaP AA cells, the AR and PSA expression levels were increased compared with those in VCaP cells. In VCaP rev as well as in VCaP AA cells, the ERβ expression was lower than in VCaP cells. The data represent two independent experiments, which were performed in duplicate. α-tubulin was used as a loading control.

We aimed to investigate the influence of an ERβ-specific ligand on a cell system that mimics the three different stages of ADT in humans. Therefore, the AR-overexpressing VCaP cells were either treated with testosterone to reset the cells to an androgen-dependent state before first-line ADT, comparable to early pre-treatment conditions, or with abiraterone to drive VCaP cells into maximal ADT, i.e., all androgen sources, including intratumoral androgen synthesis, suppressed, as in second-line ADT (Figure 1A). Both qRT-PCR (Figure 1B) and western blot analyses (Figure 1C) were performed for the three VCaP cell variants, androgen-sensitive VCaP cells (VCaP rev), VCaP cells under ADT (CRPC) and VCaP cells treated with abiraterone (VCaP AA), to confirm differential gene expression for prostate cancer-relevant genes under increasing ADT. AR mRNA expression was significantly upregulated from low levels in VCaP rev cells to VCaP cells, and AR mRNA expression was further upregulated under increasing ADT in VCaP AA cells, albeit not significantly. Similarly, AR protein expression in VCaP rev cells was downregulated by 56%, and PSA expression was reduced by 54% compared with VCaP control cells. Of note, this pretreatment condition presents with normal AR expression and no obvious PSA in the presence of low testosterone, whereas initial androgen withdrawal revealed increased castration-resistant features of AR and elevated PSA without external testosterone. Further upregulated expression of the AR (5.8-fold) and PSA expression (almost 2-fold) was observed in VCaP AA cells compared with those in untreated VCaP cells. Furthermore, splice variants of the AR (ARVs) could be detected only in VCaP AA cells concomitant with the maximum upregulation of full-length AR. qRT-PCR expression analyses revealed ARV7 as one representative ARV for the molecular structure visualized by the N-terminal AR antibody (data not shown). The western blot analysis indicates that ERβ expression was not aligned with increasing castration resistance mechanisms as demonstrated for AR, ARVs and PSA but had maximal expression in the castration-resistant pre-abiraterone stage.

Control experiments in establishing this CRPC cell model revealed VCaP cells are definitely androgen-sensitive, thus DHT (10 nM) could induce AR promoter activity. Most important for this project was, however, that there is no such activity from the compound 8β-VE2 (Supplementary Figure 1).

### Testing of the efficacy of the ERβ-specific agonist 8β-VE2

The different stages of ADT represented by VCaP rev, VCaP, and VCaP AA were used to investigate the effect of ERβ activation on increasing ADT stages. Therefore, the cells were treated with the ERβ-specific agonist 8β-VE2, and cell survival, apoptosis induction and gene expression were examined. First, different dosages of 8β-VE2 were tested in VCaP cells to determine drug efficacy (Supplementary Figure 2). Concentrations of 5 μM and 25 μM 8β-VE2 showed significant reduction of cell survival and induction of apoptosis and were used for further experiments. A concentration of 50 μM 8β-VE2 led to a diminished expression of housekeeping genes (data not shown), and were therefore excluded from further experiments.

### ERβ activation led to AR and ARV downregulation in ADT in VCaP and VCaP AA cells

**Figure 2:**
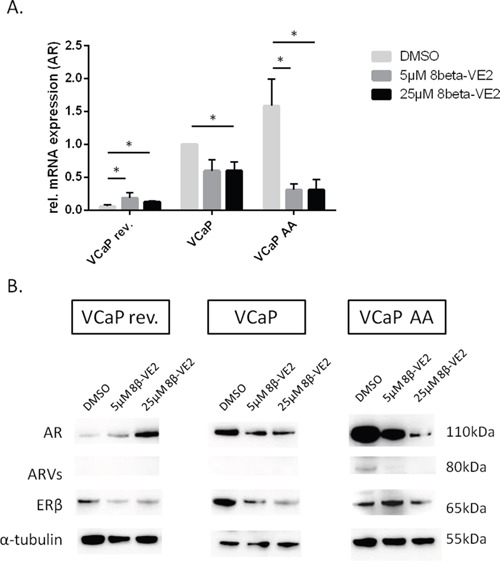
Treatments with 8β-VE2 lead to downregulation of AR and ARVs in androgen-deprived prostate cancer cells **(A)** mRNA expression of the AR was measured by quantitative RT-PCR. VCaP rev, VCaP, and VCaP AA cells were treated with DMSO, 5 μM 8β-VE2 and 25 μM 8β-VE2. AR expression was strongly reduced after treatment with 8β-VE2 in VCaP control and VCaP AA cells. In VCaP rev cells, upregulation of AR mRNA expression could be observed. The data represent the mean±s.d. of three independent experiments, which were performed in duplicate. * P<0.05, ** P<0.01, *** P<0.0001 compared with DMSO control. **(B)** A representative western blot is shown of VCaP rev, VCaP, and VCaP AA cells treated with DMSO, 5 μM 8β-VE2, or 25 μM 8β-VE2. Total protein was extracted, and immunoblots were probed with AR-specific or ERβ-specific antibodies. A reduction in protein expression of the AR was detected in VCaP and in VCaP AA cells after 8β-VE2 treatment. In 8β-VE2-treated VCaP AA cells, the expression of ARVs was remarkably downregulated. In contrast, VCaP rev cells with low initial expression showed upregulated AR expression. In all three cell populations, downregulation of ERβ protein was observed at high 8β-VE2 concentration. The data represent two independent experiments, which were performed in duplicate. α-Tubulin was used as a loading control.

Because AR signaling based on AR overexpression and the occurrence of AR splice variants was retrievable in our cell model of therapy through the sequence of intensifying ADT and is indicated by PSA progression as in clinical surveillance, we evaluated the effect of ERβ activation on AR expression. In the least malignant and androgen-sensitive stage, as indicated by low AR expression, 8β-VE2 treatment of VCaP rev cells did not further improve their low malignancy. On the contrary, especially under high concentrations of 8β-VE2, AR expression was significantly upregulated at both the mRNA and protein level (Figure 2A, 2B). Therefore, ERβ targeting as a first-line antiandrogen measure is not an option. However, in castration resistance and, most tellingly, at the brink of therapy resistance, AR mRNA was downregulated in VCaP cells by 40% and in VCaP AA cells by 70% (Figure 2A). Concordantly, AR protein expression decreased by 52% in VCaP cells and by 90% in VCaP AA cells after 8β-VE2 treatment (Figure 2B). In 8β-VE2-treated VCaP AA cells, the expression of ARVs was also remarkably downregulated by 72%. Activation of ERβ in VCaP AA cells with 5 μM 8β-VE2 already induced the desired downregulation of AR and ARVs, and further downregulation of the expression of AR and ARVs was obtained using 25 μM 8β-VE2 (Figure 2B).

### ERβ activation reduced cell survival and induced apoptosis in different VCaP cell populations

**Figure 3:**
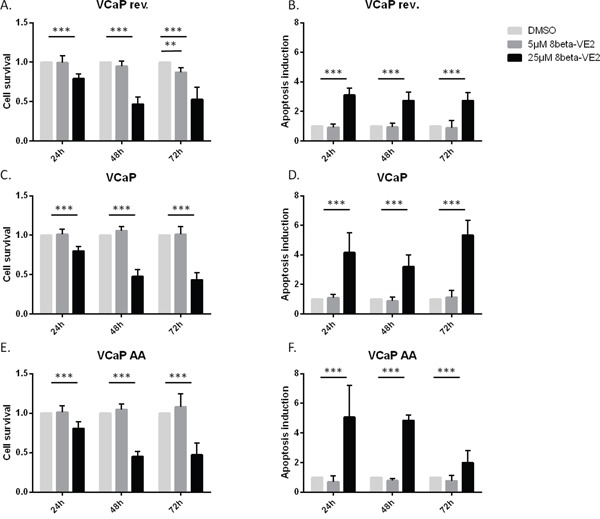
8β-VE2 reduces cell survival and induces apoptosis in different stages of prostate cancer (**A** and **B**) VCaP rev, (**C** and **D**) VCaP, and (**E** and **F**) VCaP AA cells were treated with DMSO, 5 μM 8β-VE2, or 25 μM 8β-VE2 and (**A, C, E**) cell survival and (**B, D, F**) apoptosis were measured 24, 48, and 72 h after the start of treatment. All three VCaP cell populations showed a downregulated cell survival rate compared with that of control cells. In addition, induction of apoptosis could be observed in all VCaP populations. The data represent the mean±s.d. of three independent experiments, which were performed in duplicate. * P<0.05, ** P<0.01, *** P<0.0001 compared with DMSO control.

To investigate the therapeutic potential of ERβ activation at the cellular level, both tumor cell survival and induction of apoptosis were assayed in the following experiments. The three VCaP populations (VCaP rev, VCaP, and VCaP AA) were treated with 5 and 25 μM 8β-VE2 or with DMSO as the control. Both the cell survival rate (Figure 3A, 3C, and 3E) and induction of apoptosis (Figure 3B, 3D, and 3F) were measured simultaneously using the ApoTox-Glo™ Triplex Assay. All three treated VCaP cell populations showed a significantly reduced cell survival rate of up to 60% relative to that of control cells. In addition, apoptosis was observed in all VCaP populations and was between three- and five-fold higher than that of control cells. These cell survival and apoptosis effects occurred in a time- and concentration-dependent manner and were observed in all three ADT modes. Most strikingly, tumor cell survival was lower, and apoptosis intensified faster and to the highest extent in VCaP AA cells, which are pending therapy failure; therefore, in this situation, switching therapies should be considered.

## DISCUSSION

Persistent androgen receptor signaling under sequenced therapies for metastatic castration-resistant prostate cancer (mCRPC) is the main obstacle for durable treatment benefit because therapy resistance occurs for most initially effective treatments. This statement is true even after the advent of the most effective ADTs, such as abiraterone acetate, or multifunctional new generation AR inhibitors, such as enzalutamide. Although these drugs have different molecular targets (abiraterone inhibits steroidogenesis, and enzalutamide competitively binds AR with high affinity), resistance to these therapies most likely shares a common denominator [8]. This common molecular medium of androgen-directed therapy failure most likely accounts for the phenomenon of cross resistance, i.e., effective treatments may lose much of their individual potential when applied in the second position. This molecular structure causing therapy failure for both abiraterone and enzalutamide has recently been identified potentially as constitutive active AR splice variants, represented by AR-V7 [8]. A liquid biopsy analysis including this predictive AR modification from circulating prostate cancer cells has since been established. This ability to determine key resistance-mediating AR modifications facilitates a personalized approach for decision-making in therapeutic sequences and may considerably improve prolonged prostate cancer treatment and patient survival [23].

We were able to recapitulate resistance patterns in cells from that observed after first-line ADT to complete ADT in our representative *in vitro* models of VCaP bone metastatic cells. The therapeutic sequence commenced from VCaP rev cells, which depend on the low testosterone concentration of 1 nM (0.3 ng/ml) and exhibit a normal level of AR expression (Figure 1A–1C). This cell model should represent hypogonadal therapy-naïve patients and displays androgen sensitivity. Therefore, these cells are susceptible to first-line ADT, i.e., deprivation of external androgens [19]. As observed in our cell model, first-line androgen deprivation eventually caused castration resistance (VCaP) with AR signaling depending on intratumoral steroidogenesis and AR sensitized by overexpression [2]. This cell model was treated with the clinically approved CRPC drug abiraterone to eliminate intratumoral steroidogenesis to further fortify AR overexpression (Figure 1B and 1C) and added AR splice variants (80 kDa, Figure 1C). This latter condition is considered therapy-resistant and a therapy switch from ADT to AR blockade, e.g., abiraterone to enzalutamide, is not recommended due to pending cross resistance [22, 24]. Under these AR conditions, abiraterone is no longer effective because AR splice variants are constitutively active and unreceptive to ADT, and enzalutamide cannot bind to this structure due to the lack of an AR ligand binding domain in this structure. Our model clearly shows the potential of AR splice variants to exhibit constitutive activity because the classical androgen-regulated gene PSA is expressed under complete androgen deprivation and in the absence of external androgens, i.e., virtually androgen-free PSA expression (Figure 1C).

The detection of AR splice variants by liquid biopsies for personalized therapy sequencing excluded switching to other AR-targeted therapies; thus, chemotherapy remains an option [25]. To overcome the predicament of cross resistance if CRPC therapy of next-generation ADT fails, and AR blockade by flutamide, bicalutamide or enzalutamide is less effective, we applied ERβ activation as an antiandrogen treatment using the ERβ-selective agonist 8β-VE2 [26, 17]. Although 8β-VE2 showed potential as a general prostate cancer drug by decreasing cell survival and increasing tumor cell apoptosis in all cell variants (Figure 3), there is no just cause to replace approved first-line and next-generation ADT in androgen-sensitive prostate cancer or CRPC considering the effects we obtained on the pivotal target AR (Figure 2B). However, putative therapeutic resistance from AR overexpression and splice variants can be potentially rectified with this ERβ-selective agonist (Figure 2B, VCaP AA). The reciprocal mechanism of AR/AR-Vs downregulation under ERβ activation appears to perform best with a moderate 8β-VE2 concentration of 5 μmol/L. Increased concentrations further limit AR expression but no longer on the basis of the cogent counteraction. Interesting in a mechanistic sense, the counteraction of AR and ERβ expression was demonstrated in CRPC rev cells (Figure 2B) where ERβ downregulation caused the upregulation of AR expression. This finding confirms our previous data from ERβ functional analyses with RNA interference in LNCaP cells, when ERβ knock-down caused rising PSA indistinguishable from an androgen stimulus [17]. Also in CRPC represented by VCaP cells (Figure 2B, middle) 8β-VE2 causes AR downregulation, reduced cell survival and increased apoptosis. However, this occurred only at high 8β-VE2 concentrations, probably inconsistent with therapy options (Figure 3C-3D), and not upon ERβ upregulation. Therefore, the concept of AR-counteracting ERβ seems most convincing in therapy sequencing causing cross resistance upon appearance of AR splice variants (Figure 2, right).

We estimate that the antiandrogen function of ERβ activation has considerable advantages over established antiandrogens or future concepts of AR targeting that have been considered. An enduring therapeutic benefit from established AR LBD-binding antiandrogens may be restricted due to a potential antiandrogen-to-androgen conversion by LBD gain-of-function mutations, albeit only for a minority of therapy failures compared with the AR splice variant function [5]. In contrast to established antiandrogens, the drug 8β-VE2 *a priori* activates its target ERβ; a comparable antagonist to agonist conversion due to selection of suitable AR mutant clones is therefore unlikely.

Furthermore, in the case of pre-existing gain-of-function AR mutations (e.g., T877A), the effectiveness of other, still feasible, antiandrogens (e.g., enzalutamide) for therapeutic sequencing is limited, as these AR blockers will no longer perform as true inhibitors of androgen signaling [27]. The ERβ-mediated inhibition of AR signaling by downregulation will instead be in effect regardless of gain-of-function mutations, e.g., the T877A mutation [17]. Moreover, we presume that ERβ-targeted AR inhibition is rather inoffensive, and toxic side effects may be limited compared with alternative antiandrogen considerations of AR degradation, antisense RNA-mediated AR downregulation or AR splice variant inhibition by targeting the rather unspecific target of the AR N-terminus [28–29]. Notwithstanding, an appropriate clinical application of ERβ-selective drugs depents on an arguable useful dosage. In our *in vitro* experiments best performance was revealed at 5 μM concentration which is in the same range we and others commonly use for the clinically approved drug abiraterone acetate as well as other antiandrogens [27]. *In vivo* studies with 8β-VE2 were conducted in micromolar range per kg body weigth [30–32]. A limitation to such drugs may be the ERβ-selectivity restricted to a picomolar range [33]. This would imply that the major advantage of such drugs could be a lesser affinity to the AR (Supplementary Figure 1), especially promiscuous mutant AR, as compared to estradiol [17]. Therefore, major requirement for this ERβ-mediated antiandrogen action in clinical applications will be the ER-subtype selectivity to avoid unwanted ERα activation. Interestingly, the more recently introduced ERβ-selective agonist KB9520 also increases ERβ expression in malignancies other than prostate cancer [34–35]. We anticipate a most promising application of ER-subtype selective drugs as a tool to eliminate AR splice variants and cross resistance in therapy sequences. The function of ERβ as a tumor suppressor must not be true for all cancers and is also not true for all prostate cancers [13, 36]. In AR-negative, e.g. neuroendrocrine prostate cancer a counteraction from ERβ on AR should be obsolete. Also AR-positive prostate cancers with combined mutational aberrations in the AR as in the cell modell CRW22Rv1 may be resistant to ERβ-selective treatments with 8ß-VE2. Our 8ß-VE2-treatments of such prostate cancer cells confirmed the study from Colciago et al. [37]. 8ß-VE2-treatments successful for the VCaP AA cell model (Figure 2B) failed to diminish AR expression and to eradicate AR splice variants in the CRW22Rv1 model (data not shown). Fortunately, neuroendocrine prostate cancers and refractory therapy-resistant AR mutations are rare among prostate cancer patients [23, 27]. More conflicting results referring to the general tumor suppressor function of ERβ derived from a study by Wang et al. [38]. This study revealed higher ESR2 expression in a putative marker function in tumor tissue as compared normal prostate. Therefore, future studies are warranted to substantiate defined functions of the estrogen receptors.

To our knowledge, this is the first time ERβ has been considered a targetable antiandrogen structure in the connotation of therapy failure and therapy cross resistance in AR-targeted therapies. Future research and clinical applications are necessary to determine the potential of this approach in hitherto incurable mCRPC for optimal palliative effects, prolonged survival benefits or even curable measures.

## MATERIALS AND METHODS

### Cell lines

The human prostate cancer cell line VCaP was obtained from LGC Standards (Teddington, England). VCaP revert (VCaP rev) cells were generated by treating VCaP cells with rising concentrations of testosterone up to 1 nM (Sigma-Aldrich, Taufkirchen, Germany) over a period of seven months, after which, the cells were further cultured in medium containing 1 nM testosterone [21]. VCaP AA cells were treated with increasing concentrations (up to 5 μM) of abiraterone (Janssen-Cilag, Neuss, Germany) for seven weeks and then permanently cultured in medium containing the agent.

### Cell culture

VCaP cells were cultured in phenol red-free DMEM (Life Technologies, Darmstadt, Germany) without L-glutamine or pyruvate and supplemented with 10% FBS, 1.25% penicillin/streptomycin, 5% L-glutamine (PAN Technology, Carlstadt, USA), and 2% sodium pyruvate (Life Technologies, Darmstadt, Germany). The medium for VCaP rev cells was supplemented with 1 nM testosterone. The culture medium for VCaP AA cells was supplemented with 5 μM abiraterone acetate hydrolyzed to abiraterone in ethanol/H_2_O [22]. For subsequent experiments, the cells were cultured in medium containing dextran-coated charcoal (Sigma-Aldrich, Taufkirchen, Germany)-treated FBS.

### Measurements of cell survival and apoptosis induction

For the measurement of proliferation and apoptosis, the ApoTox-Glo™ Triplex Assay (Promega, Fitchburg, USA) was used following the manufacturer's instructions. Signals were detected by the Synergy™ Mx Plate reader (BioTek, Winooski, USA). All three VCaP cell variations (2×10^4^ cells each) were cultured in 96-well plates. For pretrial experiments, concentrations between 5 and 50 μM of the ERβ-specific agonist 8β-VE2 (Bayer AG, Leverkusen, Germany) were added to the cell culture medium. DMSO (Carl Roth, Karlsruhe, Germany)-treated cells were used as a control. For further investigations, the three different VCaP cell variations were treated with either 5 or 25 μM 8β-VE2. Analyses of cell proliferation and apoptosis were performed 24, 48, and 72 h after the beginning of treatment.

### Western blot analysis

For protein isolation, 2×10^5^ cells were plated in 6-well plates. The three different VCaP cell variations were treated with either 5 or 25 μM 8β-VE2 for 72 h. Modified RIPA buffer (50 mM Tris pH 7.4, 1% NP-40, 0.25% Na-deoxycholate, 150 mM NaCl, 1 mM EDTA) was used to isolate total cell lysates. Protein concentration was determined using the Bradford assay (Nanoquant, Carl Roth, Karlsruhe, Germany). For western blot analysis, 15 μg of total cell lysates were supplemented with 4x LDS sample buffer (Life Technologies, Darmstadt, Germany) containing 10% DTT (Life Technologies, Darmstadt, Germany) and denatured at 70°C for 10 min. The probes were loaded on NuPAGE™ 4-12% Bis-Tris gels (Life Technologies, Darmstadt, Germany) in MES buffer (Life Technologies, Darmstadt, Germany). The proteins were electrotransferred to a PVDF membrane (GE Healthcare, Munich, Germany) using a semidry blotting method. The membrane was blocked in 3% BSA (Carl Roth GmbH, Karlsruhe, Germany) or in 5% dry milk (Carl Roth GmbH, Karlsruhe, Germany) in Tris-buffered saline/Tween 20. The membrane was incubated with primary antibodies against AR (1:4000, NeoMarkers, Fremont, USA, #1358), ERβ (1:10000, Cell Signaling, Danvers, USA, #5513), PSA (1:4000, Cell Signaling, Danvers, USA, #2475), or α-tubulin (1:20000, Santa Cruz Biotechnology, Heidelberg, Germany) at 4°C overnight. Subsequently, the membrane was incubated with secondary goat anti-mouse IgG-HRP or goat anti-rabbit IgG-HRP (Dianova, Hamburg, Jackson Immuno-Research, Germany) in blocking buffer for 2 h at room temperature. The proteins were visualized by enhanced chemiluminescence according to the manufacturer's instructions (ECL Prime System, GE Healthcare, Freiburg, Germany). Signals were detected using a FluorChem Q (Biozym, Hessisch Oldendorf, Germany) and analyzed using FluorChem Q SA software (Biozym Scientific GmbH).

### Real-time reverse transcription (RT)-PCR

For total RNA isolation, 2×10^5^ cells were plated in 6-well plates. The three different VCaP cell variants were treated with either 5 or 25 μM 8β-VE2 for 72 h. Total RNA was isolated using the PeqGold Total RNA Kit (Peqlab, Erlangen, Germany). RNA concentrations were determined using a Nanodrop 2000C spectrophotometer (Thermo Scientific, Waltham, USA). Reverse transcription of the RNA was performed using SuperScript® II Reverse Transcriptase (Life Technologies, Darmstadt, Germany) cDNA polymerase; 1 μg of RNA was used for the reaction. As a control for efficient cDNA synthesis, PCR with glyceraldehyde 3-phosphate dehydrogenase (GAPDH)-specific primers (fw-CCAGCAAGAGCACAAGAGGAAGAG; rev-AGCACAGGGATACTTTATTAGATG) was performed. For the real-time RT-PCR analysis, 40 ng of cDNA was used as template. Platinum® SYBR® Green qPCR SuperMix-UDG with ROX (Life Technologies, Darmstadt, Germany) was used for signal detection. The threshold cycle (CT) values for AR expression (fw-AGGAACTCGATCGTATCATTGC, rev-CTCTGCCATCATTTCCGGAA) were normalized to expression levels of the housekeeping genes hypoxanthine phosphoribosyltransferase (HPRT) (fw-ACCCTTTCCAAATCCTCAGC, rev-GTTATGGCGACCCGCAG) and lactate dehydrogenase A (LDHA) (fw-GGAGATCCATCATCTCTCCC, rev-GGCCTGTGCCATCAGTATCT). Signal detection was performed using a 7900HT sequence detection system (Applied Biosystems, Darmstadt, Germany), and data were analyzed using SDS 2.3 software.

## SUPPLEMENTARY MATERIALS FIGURES AND TABLES


