# Supplementary material for: Prospects of estrogen receptor β activation in the treatment of castration-resistant prostate cancer
Source: Oncotarget. 2017 Mar 23;8(21):34971–9. doi: 10.18632/oncotarget.16496 (PMC5471027; doi:10.18632/oncotarget.16496)
Supplement: Supplementary file 1 [file oncotarget-08-34971-s001.pdf]

Prospects of estrogen receptor β activation in the treatment of castration-resistant prostate cancer

SUPPLEMENTARY MATERIALS AND METHODS

In order to measure the activity of transcription factors, the reporter system Dual-Luciferase Reporter Assay (Promega, Fitchburg, WI, USA) was used. Here, 6x 10^4 VCaP cells each were transfected with reporter vectors for either steroid receptor (MMTV-Luc, kindly provided by Megan Cully, Cancer Research, UK, [33]), or AR (rPb-Luc, pGL3-basic carrying the minimal rat probasin promoter, [34]) induced transcription and a PRL-Luc vector (Promega, Fitchburg, WI, USA) as reference for transfection efficiency. 24h after transfection, cells were stimulated with 10nM DHT, 25µM 8β-VE2, or DMSO as control and incubated for another 24h. VCaP cells were lysated according to the manufacturer's instructions and luciferase activity was measured using the Synergy Mx plate reader.

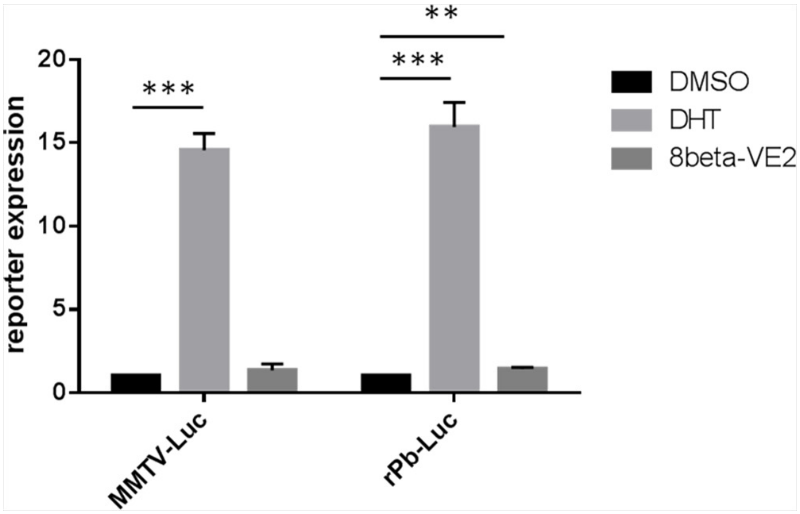

Supplementary Figure 1: In order to prove that 8β-VE2 treatment did not induce unspecific AR activation a reporter assay was performed. Transcriptional AR activity in DHT and 8β-VE2 treated VCaP cells was quantified. VCaP cells were treated with DMSO, 10nM DHT, or 25µM 8β-VE2 and MMTV and rPb promoter activation was measured 24h after treatment start. DHT treatment strongly induced both MMTV and rPb regulated luciferase (Luc) reporter gene expression. Treatment with 8β-VE2 did not activate the MMTV promoter at all and only minor rPb regulated reporter gene expression was induced by 8β-VE2. Data represent the mean +/- s.d. of three independent experiments, which were performed in duplicates. * $P<0.05$, ** $P< 0.01$, *** $P<0.0001$ compared to DMSO control.

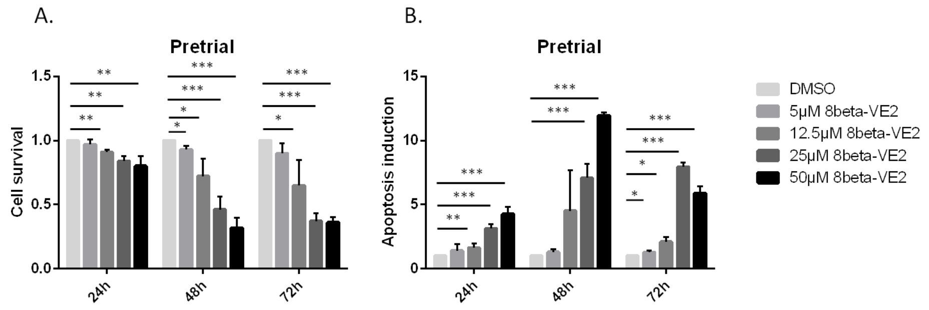

Supplementary Figure 2: Efficacy evaluation of ERβ-specific agonist 8β-VE2. VCaP cells were treated with DMSO, 5 μM 8β-VE2, or 25 μM β-VE2 and (**A**) cell survival and (**B**) apoptosis were measured 24, 48, and 72 h after the start of treatment. The data represent the mean±s.d. of three independent experiments, which were performed in duplicate. * $P<0.05$, ** $P<0.01$, *** $P<0.0001$ compared with DMSO control.
